# Humidity-Dependent Bacterial Cells Functional Morphometry Investigations Using Atomic Force Microscope

**DOI:** 10.1155/2010/704170

**Published:** 2010-06-20

**Authors:** Hike Nikiyan, Alexey Vasilchenko, Dmitry Deryabin

**Affiliations:** ^1^Department of Biochemical Physics, Orenburg State University, Pobedy Avenue, 13, Orenburg 460018, Russia; ^2^Department of Microbiology, Orenburg State University, Pobedy Avenue, 13, Orenburg 460018, Russia

## Abstract

The effect of a relative humidity (RH) in a range of 93–65% on morphological and elastic properties of *Bacillus cereus* and *Escherichia coli* cells was evaluated using atomic force microscopy. It is shown that gradual dehumidification of bacteria environment has no significant effect on cell dimensional features and considerably decreases them only at 65% RH. The increasing of the bacteria cell wall roughness and elasticity occurs at the same time. Observed changes indicate that morphological properties of *B. cereus* are rather stable in wide range of relative humidity, whereas *E. coli* are more sensitive to drying, significantly increasing roughness and stiffness parameters at RH ≤ 84% RH. It is discussed the dependence of the response features on differences in cell wall structure of gram-positive and gram-negative bacterial cells.

## 1. Introduction

Significant progress in using of atomic force microscope (AFM) as a tool for investigations of eukaryotic and prokaryotic cells has been reached during past decade [1, 2]. In contrast to traditional methods of visualization—scanning electron and optical microscopy—atomic force microscopy offers important benefits: high spatial resolution, real quantitative data acquisition in three dimensions, relatively simple and nondestructive sample preparation procedure, and flexibility in ambient operating conditions (i.e., without the need for a vacuum or gold sputtering) [3]. Besides topographic imaging, AFM makes it possible to probe local surface forces and mechanical properties of biomaterials [4]. In particular, mechanical properties of mammalian [5] and bacterial cells [6] have been measured.

Though the method of atomic force microscopy is relatively new, it could become widespread in microbiological studies that use bacteria as sensors, changing their morphological characteristics at various exposures. Thus, AFM has been used to study temperature-dependent morphological alterations of prokaryotic cells [7] and effects of antibiotics on E. coli and S. aureus [8].

It is important to take into consideration that different environmental conditions that often remain unregistered could distort AFM results at investigation of physical and morphological properties of bacterial cells. For example, the humidity of the environment where AFM specimens are left to dry is often ignored [8, 9], though distinct differences in morphology of bacterial cells growing at different relative humidity were observed by De Goffau et al. [10]. Therefore, the development and standardization of AFM methods for preparation and imaging of bacterial cells in different environmental conditions are of great importance for microbiology. The standardization of the methods will allow to compare results, obtained by different authors, and is an essential condition for carrying out multicentre studies.

The aim of this study was to evaluate the effect of a relative humidity on results of AFM investigation of morphological characteristics and mechanical properties of *Bacillus cereus* and *Escherichia coli* bacteria.

## 2. Materials and Methods

Gram-negative *E. coli K12 *and gram-positive *B. cereus ip5832* strains were used as model organisms to investigate the effects of the relative humidity on the morphology of the cells. Bacteria were grown in 2.5 ml nutrient bouillon (GRM-bouillon contains pancreatic digest of fish flour and sodium chloride, pH 7.0) at 37°C for 24 hours to produce a bacterial concentration of *≈*10^9^ viable cells ml^−1^. Bacterial concentrations were determined by measuring A540 of the culture in a spectrophotometer (SF-46, LOMO, Russia). Samples of each strain were collected, centrifuged (3000 rpm, 1700 g, 7 min) and twice washed with distilled water. The drop of the cell suspension was deposited then on pieces of freshly cleaved mica (5 × 5 mm) which were placed for 24 hours in different exsiccators to dry. To maintain required ambient humidity, saturated aqueous salt solution was also placed in the exsiccators. Percentage of relative humidity (RH) above saturated solutions of used salts at 25°C according to Greenspan [11] was 65% (NaNO_2_), 75% (NaCl), 84% (KCl), and 93% (KNO_3_).

Bacteria were imaged in contact mode, using SMM-2000 AFM (JSK “KPD”, Russia). Images were obtained using V-shaped silicon nitride cantilevers MSCT-AUNM from Veeco Instruments Inc. with a spring constant of 0.01 N/m. 

Root-mean-square roughness (the standard deviation of the *Z *values) for the height images was determined by drawing section plot of the cell surface and was calculated using SMM-2000 software. The images were flattened and plane fitted prior to analysis. The reliability of the difference was estimated according to nonparametric Wilcoxon's signed-rank test. 

After the determination of the bacteria surface topography, force curves were obtained at various locations in each cell. Force-indentation curves were derived from the measured force versus displacement relationship using the mica surface to calibrate the deflection of the cantilever. To obtain Young's modulus of the cell we used the Hertz model [12]. Equation (1) shows the relationship between the load force (*F*) and the sample indentation (*δ*):

(1) $$F=\frac{4}{3}\cdot \frac{E}{1-\nu^{2}}\cdot \delta^{3/2}\cdot \sqrt{R},$$

where *E* is Young's modulus, *R* is the probe-sphere radius, and *ν* is the Poisson ratio. The Poisson ratio *ν* of the cells was chosen to be 0.5. The sample indentation *δ* is calculated by subtracting the piezo displacement from the cantilever deflection. Young's modulus calculation procedure from force-indentation relations is described in [13].

## 3. Results and Discussion

**Figure 1:**
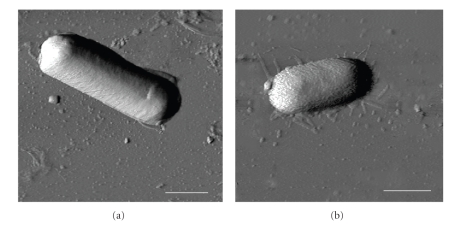
AFM phase images of *B. cereus* (a) and *E. coli* (b) on mica surface at 93% RH. The white bar indicates 1 *μ*m. There are no qualitative changes that can be seen in the pictures at different RH values.

Typical AFM images of *B. cereus* and *E. coli* on mica surface are shown in Figure 1. For each type of cells, the following morphological parameters were measured: length, width, and height. Relying on this data, perimeter section, area section, and volume of the cells were calculated. At least 30 cells were processed to calculate mean values for each parameter. The mean values of mentioned parameters are presented in Table 1. 

The observed changes of morphological parameters should also affect morphological properties of a surface. To reveal such changes, the surface roughness analysis and determination of bacterial cell elasticity were performed for each grade of dehumidification.

**Figure 2:**
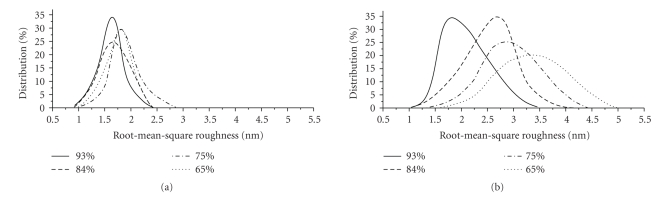
Roughness distribution of *B. cereus* (a) and *E. coli* (b).

Root-mean-square roughness (*R*_*q*_) distribution of studied structures is shown in Figure 2. There is a gradual increase of cell wall roughness taking place at RH reduction both for *B. cereus* and for* E. coli.*

The shift of RH from 93% to 65% leads to the change of roughness of *B. cereus* surface insignificantly (Figure 2(a)). At 93% the mean value of roughness has 1.6 nm and a symmetrical character of roughness distribution is observed. Insignificant changes of roughness occur at decreasing of humidity down to 65%: the *R*_*q*_ values in this case are within 1.6–1.9 nm range. The roughness distribution curves during the dehumidification are shifting in direction of greater roughness values that describe the reaction of gram-positive bacteria at RH reduction in general. 

In comparison with *B. cereus*, cell surface of gram-negative *E. coli* is more rough (Figure 2(b)). The average value of *R*_*q*_ parameter at 93% RH is 1.7 nm. The transition from 93% to 65% RH is accompanied by more evident changes in cell surface, as compared to *B. cereus*. Reduction in RH leads to increasing of the cells roughness (*R*_*q*_ = 3.4 nm). The symmetry of the cells distribution is characterized by the shift and tilt in the direction of greater roughness values. Roughness distribution curves illustrated in Figure 2(b) show that the dehydration of *E. coli* bacteria, as in the case of *B. cereus*, is a gradual process. 

**Figure 3:**
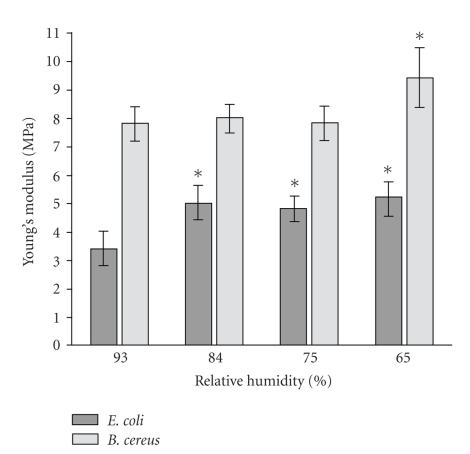
Diagram of Young's modulus distribution; *−*P* < .05 (Wilcoxon's signed-rank test).

The distribution diagram of Young's modulus on RH is shown in Figure 3. Force curves were obtained from at least 30 bacterial cells for each RH value. Additional measurements from various locations of the bacteria surface for each cell were made. The diagram shows that reduction of RH causes increase of the two bacteria stiffness. Reliable changes of *B. cereus* stiffness are noticed only at minimal values of RH—65%. Quite contrary behavior of stiffness change is demonstrated by *E. coli* cells—the fall of humidity on 10% causes the increase of Young's modulus from 3.4 to 5 MPa. As can be seen in diagram, further stiffness growing is insignificant. 

Discussing the obtained results, we have determined that the gradual dehumidification of the gram-positive *B. cereus* cells environment does not lead to significant changes of morphological parameters, but induces increase of surface roughness and cell wall stiffness. All studied characteristics remain stable in wide range of RH, partly changing at reaching 65% RH. Fixed changes included decreasing of “width” parameter and increasing of cell wall stiffness as a result of dehumidification. The roughness of bacteria during the humidity reduction undergoes reliable changes only at 65% and amounted to 1.9 nm, whereas at 93% it was 1.6 nm. In comparison with gram-negative *E. coli*, cell wall of *B. cereus* is more rigid and demonstrates high resistance to humidity drop. Reliable changes of bacteria stiffness occur also at 65% RH. Quantitative changes are expressed in growth of Young's modulus from 7.9 MPa to 9.4 MPa. 

More significant changes occur with gram-negative *E. coli* during dehumidification; however, behavior of the changes is different. *E. coli* bacteria are more sensitive to humidity difference: reliable changes of the roughness are already detected at reduction of RH from 93% (*R*_*q*_ = 1.7 nm) to 84% (*R*_*q*_ = 2.6 nm). The further humidity fall is accompanied by growth of a roughness up to 3.4 nm at 65%. At a humidity transition from 93% to 84%, the loss of mechanical properties of *E. coli* cellular wall occurs; it is expressed by shifting of Young's modulus values from 3.4 MPa to 5 MPa.

We suppose that the described differences in surface and elastic properties are in distinction of cell wall structure of gram-negative and gram-positive bacteria. Gram-positive bacteria possess a thick cell wall containing many layers of peptidoglycan and teichoic acids and are therefore initially more rigid [14]. Such structure allows to retain water inside the cell at RH values over 65% and demonstrates high tolerance of bacteria to drying. From our point of view, this resistance to drying reflects features of cell wall molecular organization of *B. cereus* and other gram-positive microorganisms that have three-dimensional bag-shaped peptidoglycan sacculus [15]. Such organization of a cell wall provides possibility of microorganisms' existence in natural habitats where differences of relative humidity can be essential. 

Contrary to them gram-negative cells have a relatively thin cell wall [14] and reveal lesser resistance to RH change; therefore, at 84% *E. coli* cells already lose their mechanical strength and significantly increase the roughness of the surface. This process can be explained by rearranging of liposaccharides and other membrane components that have to occupy smaller area of cell surface at dehumidification. 

Our results explain processes occurring with bacteria at native environment and may be useful for standardization of conditions at morphometry of the biological objects. Therefore, during specimen preparation and scanning, it is recommended to sustain ambient humidity for *E. coli* and other gram-negative bacteria on the level of 93%. For *B. cereus* and other gram-positive bacteria RH values may lay within a range of 93%–75%.

## 4. Conclusions

The effect of a relative humidity on morphological and elastic properties of *Bacillus cereus* and *Escherichia coli* cells is evaluated using atomic force microscopy. It is determined that such morphological characteristics as length, width, height, and cell volume are relatively stable at drying. On the other hand, roughness of a bacterial surface and especially stiffness of the cell significantly increase at dehumidification. It is shown a dependence of registered changes on cell wall structure of model bacteria. Gram-positive *B. cereus* cells change morphological and mechanical properties only at 65% RH, whereas gram-negative *E. coli* are more sensitive, significantly increasing their parameters at RH ≤ 84% RH. These findings can explain some ecology features of the bacteria, defining requirements to RH standardization at study of different groups of microorganisms.

## Figures and Tables

**Table 1:** Morphological characteristics of *B. cereus* and *E. coli. *

Strain	RH (%)	Morphological characteristics					
		Length min-max (*μ*m)	Width min-max (*μ*m)	Height min-max (*μ*m)	Perimeter section (*μ*m)	Area section (*μ*m²)	Volume (*μ*m^3^)
B. cereusip5832	93	2.97 ± 0.51	1.08 ± 0.10	0.28 ± 0.03	2.14 ± 0.19	0.24 ± 0.04	0.71 ± 0.19
		2.03−3.88	0.87−1.26	0.24−0.35			
	84	2.93 ± 0.32	1.09 ± 0.11	0.28 ± 0.02	2.15 ± 0.16	0.24±0.03	0.70 ± 0.14
		2.36−3.59	0.88−1.30	0.23−0.31			
	75	2.89 ± 0.51	1.04 ± 0.05	0.27 ± 0.27	2.06 ± 0.11	0.22 ± 0.03	0.64 ± 0.13
		2.03−3.82	0.95−1.14	0.22−0.32			
	65	2.85 ± 0.60	0.96 ± 0.12**	0.27 ± 0.36	1.93 ± 0.20**	0.20 ± 0.04**	0.58 ± 0.18
		1.54−4.16	0.69−1.19	0.16−0.32			
E. coli K12	93	2.46 ± 0.35	1.25 ± 0.26	0.20 ± 0.02	2.28 ± 0.40	0.20 ± 0.04	0.48 ± 0.14
		2.03−3.38	0.77−1.59	0.15−0.22			
	84	2.13 ± 0.33	1.27 ± 0.12	0.20 ± 0.03	2.31 ± 0.19	0.20 ± 0.03	0.42 ± 0.12
		1.50−2.82	1.10−1.46	0.15−0.24			
	75	2.35 ± 0.40	1.27 ± 0.17	0.20 ± 0.03	2.31 ± 0.27	0.20 ± 0.04	0.47 ± 0.11
		1.65−2.99	0.96−1.58	0.15−0.25			
	65	2.30 ± 0.57	1.10 ± 0.19*	0.21 ± 0.04	2.06 ± 0.32	0.18 ± 0.05	0.42 ± 0.18
		1.49−3.54	0.71−1.48	0.14−0.27			

*−*P* < .05; **−  *P* < .01 (Wilcoxon's signed-rank test).

## References

[1] Gebeshuber IC, Smith RAP, Winter HP, Aumayr F, Evangelista V, Barsanti L, Passarelli V, Gualtieri P (2005). Scanning probe microscopy across dimensions. *From Cells to Proteins: Imaging Nature across Dimensions, Proceedings of the NATO Advanced Study Institute, Pisa, Italy, September 2004*.

[2] Dufrêne YF (2002). Atomic force microscopy, a powerful tool in microbiology. *Journal of Bacteriology*.

[3] Braga PC, Ricci D (2000). Detection of rokitamycin-induced morphostructural alterations in Helicobacter pylori by atomic force microscopy. *Chemotherapy*.

[4] Tao NJ, Lindsay SM, Lees S (1992). Measuring the microelastic properties of biological material. *Biophysical Journal*.

[5] Radmacher M, Fritz M, Kacher CM, Cleveland JP, Hansma PK (1996). Measuring the viscoelastic properties of human platelets with the atomic force microscope. *Biophysical Journal*.

[6] Arnoldi M, Fritz M, Bäuerlein E, Radmacher M, Sackmann E, Boulbitch A (2000). Bacterial turgor pressure can be measured by atomic force microscopy. *Physical Review E*.

[7] Guschina YuYu, Olyunina LN, Goncharova TA, Veselov AP, Matskova YuA, Ezhevskaya MA (2005). Investigation of Azotobacter chroococcum surface morphology in hyperthermia environment by method of atomic force microscopy. *Journal of Surface. Roentgen, Synchrotron and Neutron Studies*.

[8] Perry CC, Weatherly M, Beale T, Randriamahefa A (2009). Atomic forcemicroscopy study of the antimicrobial activity of aqueous garlic versus ampicillin against Escherichia coli and Staphylococcus aureus. *Journal of the Science of Food and Agriculture*.

[9] El Kirat K, Burton I, Dupres V, Dufrene YF (2005). Sample preparation procedures for biological atomic force microscopy. *Journal of Microscopy*.

[10] De Goffau MC, Yang X, Van Dijl JM, Harmsen HJM (2009). Bacterial pleomorphism and competition in a relative humidity gradient. *Environmental Microbiology*.

[11] Greenspan L (1977). Humidity fixed points of binary saturated aqueous solutions. *Journal of Research of the National Bureau of Standards. Section A*.

[12] Hertz H (1881). Über die Beruhrung Fester Elastischer Korper (On the Contact of Elastic Solids). *Journal für die reine und angewandte Mathematik*.

[13] Salerno M, Bykov I (2006). Tutorial: mapping adhesion forces and calculating elasticity in contact-mode AFM. *Microscopy and Analysis*.

[14] Lerner KL, Lerner BW (2003). Bacterial membranes and cellwall. *World of Microbiology and Immunology*.

[15] Ghuysen JM, Hakenbeck R (1994). *Bacterial Cell Wall*.

